# “When I’m in Pain, Everything Is Overwhelming”: Implications of Pain in Adults With Autism on Their Daily Living and Participation

**DOI:** 10.3389/fpsyg.2022.911756

**Published:** 2022-06-14

**Authors:** Merry Kalingel-Levi, Naomi Schreuer, Yelena Granovsky, Tami Bar-Shalita, Irit Weissman-Fogel, Tseela Hoffman, Eynat Gal

**Affiliations:** ^1^Department of Occupational Therapy, Faculty of Social Welfare and Health Sciences, University of Haifa, Haifa, Israel; ^2^Laboratory of Clinical Neurophysiology, Bruce Rappaport Faculty of Medicine, Technion, Israel Institute of Technology, Haifa, Israel; ^3^Department of Neurology, Rambam Health Care Center, Haifa, Israel; ^4^Department of Occupational Therapy, School of Health Professions, Faculty of Medicine, Tel Aviv University, Tel Aviv, Israel; ^5^Department of Physiotherapy, Faculty of Social Welfare and Health Sciences, University of Haifa, Haifa, Israel

**Keywords:** pain sensitivity, pain processing, pain awareness, coping strategy, function

## Abstract

Pain sensation in autism spectrum disorder (ASD) has been a growing research field in the last two decades. Existing pain research has focused on pain sensitivity, suggesting either hyposensitivity or hypersensitivity to pain in individuals with ASD. However, research about other aspects of pain experience is scarce. Moreover, most pain-related research in ASD focused on quantitative measures, such as neuroimaging or parental reports. Instead, this paper aimed to illuminate the various aspects of pain experience as perceived by adults with ASD. Its descriptive qualitative research design incorporated semi-structured interviews and deductive thematic analysis. This phenomenological approach captured the subjective pain experience through the lens of people with ASD. Four primary themes emerged from the data: (a) physical pain experience, including the sequence of pain sensitivity, pain awareness, pain-related emotional aspects, and pain communication; (b) direct and indirect coping strategies; (c) function and participation outcomes; and (d) suggestions for Healthcare Providers. The findings echo the crucial role of pain awareness and communication in the pain experience of people with ASD. These two factors have been reported as profoundly influencing coping strategies, function, and participation. The results emphasize the need to expand the exploration of pain in this population, calling for greater understanding, and listening to this population’s unique pain profiles and experiences to promote better-suited evaluation, diagnosis, and intervention in pain conditions.

## Introduction

Autism spectrum disorder (ASD) refers to complex neurobehavioral and neurodevelopmental conditions characterized by persistent deficits in social interaction and communication and restricted and repetitive patterns of behavior or interests. The onset of these symptoms appears in the early developmental period and results in impaired daily functioning (Tantam, 2012; American Psychiatric Association, 2013).

The *Diagnostic and Statistical Manual of Mental Health Disorders* (5th ed.; *DSM-5*) definition of ASD by American Psychiatric Association (2013) includes sensory features as one of four restricted/repetitive behavior diagnostic criteria. It defined *sensory features* as “hyper- or hypo-reactivity to sensory input or unusual interest in sensory aspects of the environment” (50). Sensory features refer to patterns of behavior suggesting differences in how daily sensory stimuli are processed (Schaaf and Lane, 2015). They may manifest differently across individuals with ASD and across time and contexts for the same person. These differences presumably result from individual capacities combined with challenges encountered in complex physical and social environments (Baranek et al., 2006).

Sensory feature differences may manifest in sensory thresholds and self-regulation strategies. Sensory thresholds range from high (slow to detect) to low (quick to detect); self-regulation strategies range from passive (not troubled by sensory stimuli) to active (reactive to sensory stimuli). Such sensory profiles characterize every human being and are conceptualized as continua (Dunn, 2014). However, *sensory atypicalities* characterize only some individuals and are more common among individuals with ASD (Little et al., 2018).

Sensory features have been noted among individuals with ASD since the earliest recorded case studies. The descriptions of Kanner (1943) included pleasure and enjoyment in the presence of sensory stimuli and increased sensory sensitivity to other stimuli. Asperger (1944) also described children with ASD demonstrating hypersensitivity in some circumstances but ignoring or seeking specific stimuli in other situations.

The two most common behavioral manifestations of sensory atypicalities are hypo- and hyperresponsiveness to sensory input, with probable differing response profiles across and within sensory modalities (Baranek, 2002; Tavassoli et al., 2018). According to Miller et al. (2007) sensory processing model, low sensory thresholds characterize individuals who cope with sensory overresponsivity. These individuals respond to sensation with more intensity or for longer durations than do those with typical sensory responsivity. Hence, they represent a *sensory-sensitive* sensory profile. Individuals who cope with high thresholds are divided into *sensory under-responsivity* and *sensory seeking* subgroups. The first group disregards or does not respond to sensory stimuli in their environment and appears not to detect incoming sensory information. The second group craves an unusual amount or type of sensory input and seems to have an insatiable desire for sensation. Atypical sensory features negatively affect the ability to respond adaptively to environmental demands and engage meaningfully in daily occupations (Dunn, 2007; Hochhauser and Engel-Yeger, 2010; Ricon et al., 2017).

Pain is explained commonly in terms of the perceptual activity of a distinct sensory modality. However, some theoreticians claim that pain is a functionally integrated feature of all the senses rather than a distinct sensory modality (Gray, 2014) or attribute pain to other sensory modalities, such as interoception (Craig, 2002).

*Nociception* is a particular type of pain processing, defined as the encoding and processing of noxious stimuli due to nociceptor activation (Garland, 2012). *Pain threshold* is defined as the amount of time elapsed before the participant reports the stimulus to be painful. The pain threshold is relatively consistent for each person under a given stimulus (Kanner, 2009). Scholars suggested that pain is not only physiological by nature but also a multidimensional phenomenon. In addition to its sensory aspect, pain involves cognitive, motivational, and affective qualities and affects function, quality of life, and well-being (Craig, 2009; Love-Jones, 2019). *Pain* is an unpleasant sensory and emotional experience associated with or resembling that associated with actual or potential tissue damage (Raja et al., 2020). *Pain processing* is the way that the brain recognizes and interprets pain (Garland, 2012).

The neurotypical population also includes individuals with under-responsiveness and over-responsiveness to pain (Bar-Shalita et al., 2019). Research indicated that individuals without developmental disorders and with sensory over-responsiveness demonstrated hypersensitivity to daily painful events and experimental pain, and experience longer pain aftersensation (Bar-Shalita et al., 2014).

Pain research in ASD is scarce and insufficient. The prevailing assumption is that individuals with ASD are hyposensitive to pain (Moore, 2015). Even in the *DSM-5*’s ASD definition, the sensory features component includes “an apparent indifference to pain/temperature” (American Psychiatric Association, 2013, p. 50). In the last decade, the comprehensive analyses of Moore (2015) and Allely (2013) focused on physical pain among individuals with ASD. Moore (2015) concluded that individuals with ASD do not present abnormal pain responses, whereas Allely (2013) acknowledged the presence of pain hyposensitivity in case studies but concluded that physiological reports challenge the ASD pain-hyposensitivity dogma. Those reviews demonstrated the scarcity of knowledge regarding pain perception features. The ASD features, including challenges in social communication and sensory atypicalities, may directly influence the experience and expression of pain (Nader et al., 2004). Those possible influences may affect pain assessment in daily life and, in turn, the quality of care that people with ASD receive (Allely, 2013; Moore, 2015). A better understanding of pain experience features among individuals with ASD is crucial because pain may significantly affect their function, participation, and quality of life.

Over the past 50 years, sensory sensitivity, sensory overload, and perceptual distortions have been reported extensively in autobiographical accounts by individuals with ASD (e.g., Grandin, 1992). They often described overwhelming sensory input as an impetus for social withdrawal (e.g., Grandin, 1996). Several autobiographical accounts focused on sensory experiences (e.g., Elwin et al., 2012) but not on pain specifically. Only one qualitative research focused on the pain experience among individuals with ASD. It indicated that adults with ASD often feel pain differently than neurotypical individuals, and some reported experiencing pain evoked by stimuli that usually do not cause pain in neurotypical adults. In addition, it suggested that pain could hinder individuals’ participation in daily occupations and risk their safety (Kornblau et al., 2020).

Considering the significant effects of pain on the lives of individuals with ASD, there is a need to characterize and better understand how pain influences their function, how they cope with it, and what they require for appropriate care. Therefore, this study aimed to shed light on how adults with ASD experience and perceive pain, its consequences on their daily living, and their pain-related coping strategies.

## Materials and Methods

This study is a part of a concurrent parallel mixed methods research (Creswell and Plano Clark, 2011), which included an inclusive, comprehensive quantitative research project and a qualitative study. All interviewees participated in the quantitative research and were purposefully invited to participate in the qualitative research. The current paper addresses the qualitative research only.

This qualitative study used a phenomenological approach to explore the study objectives. This approach is suitable for examining perspectives on complex, ambiguous, and emotionally laden issues. Pain is a prime exemplar of such a phenomenon because it is elusive, involves complex psychosomatic interactions, and is difficult to articulate (Smith and Osborn, 2015).

### Participants

We used purposeful and criterion sampling to select participants who could relate to and describe the pain experience (Creswell and Poth, 2017). Inclusion criteria were as: (a) adults with Level 1 ASD, as diagnosed using the Autism Diagnostic Observation Schedule™ (2nd ed.; Lord et al., 2012); (b) verbal performance and full-scale estimate of 80 and above on the Wechsler Abbreviated Scale of Intelligence-II (Wechsler, 2011); and (c) proficiency in the Hebrew language with an ability to provide in-depth descriptions of life experiences. The exclusion criterion was any chronic pain diagnosis.

The sample comprised 15 adults with ASD: seven males and eight females. This sample is not representative of the larger populace diagnosed with ASD, in which the female-to-male ratio is 1:4 (Maenner et al., 2020). We chose a larger representation of women with ASD since they proved more willing to share their experiences and demonstrated the ability to provide deep and rich descriptions and insights. Moreover, the larger representation of women in this study enables echoing the underrepresented population in previous literature. The participants’ mean age was 28.27 years (*SD* = 6.95). None had chronic neurological diseases, but three (20%) coped with chronic health conditions that were not pain-related. Two (13.3%) participants had been involved in work/automobile accidents, one (6.6%) experienced a head injury, and four (26.6%) experienced serious upper- or lower-extremity injuries. Nine (60%) participants used medicines, including antidepressants, anxiolytics, antipsychotics, or chronic disease medications. Table 1 presents additional personal characteristics by participant.

**Table 1:** Participants’ personal characteristics.

Participant (pseudonym)	Sex	Age	Family status	Housing accommodation^a^	Occupation
AA	M	29	Single	Apartment in community	Unemployed
AC	M	23	Single	With family members	Student
AD	M	25	Single	Independent living	Musician
AK	M	36	Single	Apartment in community	Integrated circuit designer
BF	F	46	Married	Independent living	Office assistant
BG	M	22	Single	Hostel	Unemployed
EK	F	40	Married	Independent living	Research assistant
GM	F	23	Single	With family members	Unemployed
NB	M	29	Single	Apartment in community	Geographic information systems specialist
NC	F	29	Single	With family members	Jeweler
OK	F	23	Single	Apartment in community	Unemployed
OL	M	22	Single	Hostel	Unemployed
OM	F	29	Single	Apartment in community	Office assistant
RS	M	25	Single	Apartment in community	Student
TC	F	23	Single	Hostel	Janitor

Hostel: arrangement for people with autism who can use community services and be involved in the community; Apartment in community: accommodation for those who can function independently but need support and direction in their independent lives; and Independent living: housing with no external assistance, alone, or with family members (partner, offspring, etc.).

a With family members: live with parents or other family members (grandparents, siblings, etc.).

### Procedures

The University of Haifa Ethics Committee of the Faculty of Social Welfare and Health Sciences approved this study, and all participants signed an online informed consent form. We assured participants’ anonymity and confidentiality by coding and removing their identifying details and allowing them to withdraw from the study at any stage.

Participants were invited to online, face-to-face, in-depth interviews using Zoom videoconferencing software. Each interview lasted 60–90 min and was audio-recorded only. The first author, a trained occupational therapist experienced in working with adults with ASD, conducted the interviews sensitively and facilitated open and safe communication with the participants.

The interviews were adapted to people with ASD by, for example, a preliminary conversation regarding their preferred setting; editing the interview guide while considering simple and concrete language; inviting the participants to ask for clarifications and breaks; reminding them of the option to avoid unpleasant questions; and conducting two follow-up telephone conversations to make sure that they were well after the interview.

### Research Tools

We developed an interview guide based on the literature, preliminary results of earlier quantitative research on pain among adults with ASD, and clinical experience. The questions in the interview guide referred to various issues relevant to the pain experience (e.g., subjective definition of pain, pain triggers, changes regarding pain throughout the lifespan, implications of pain on daily function, and strategies to cope with pain). The open-ended questions provided a flexible framework that invited interviewees to lead the interview according to their perspectives while enabling the researcher to maintain conceptual and structural focus on the relevant issues studied among all interviewees.

### Data Analysis

We analyzed the interviews by examining thematic content based on the participants’ descriptions of their experiences, feelings, thoughts, and perceptions. The analytic process consisted of three stages (Corbin and Strauss, 2015): (1) initial analysis, in which interviews quotations formed textual meaning units and were grouped into initial categories; (2) mapping analysis, which revealed similarities and divergence among the interviewees, potential meanings, examples, perspectives, and best practices; and (3) focused analysis, in which we conceptualized the findings into four major themes.

We stopped analyzing the interviews at participant 15 because we recognized content saturation without new information or themes emerging from the data (Charmaz, 2014).

Initially, the first two and the last authors coded the first three interviews independently, resulting in 42 preliminary categories. They then merged them into 21 final categories through deliberation and arriving at a consensus. These categories were then cautiously mapping the remaining interviews in two brainstorming sessions. The authors validated each category while considering demonstrative quotations from the data. This process resulted in conceptualizing the four main themes and creating a visual model. The whole process was presented to five professionals in the field of pain and ASD, who confirmed the analysis.

The participants’ thick and rich descriptions, demographic data related to pain, and contexts ensured the study’s trustworthiness. In addition, open conceptual discussions were conducted continually alongside peer-reviewed data processing and the authors’ familiarity with the examined phenomena. Comparing the findings with the limited literature available further enhanced the study’s credibility.

## Results

**Figure 1:**
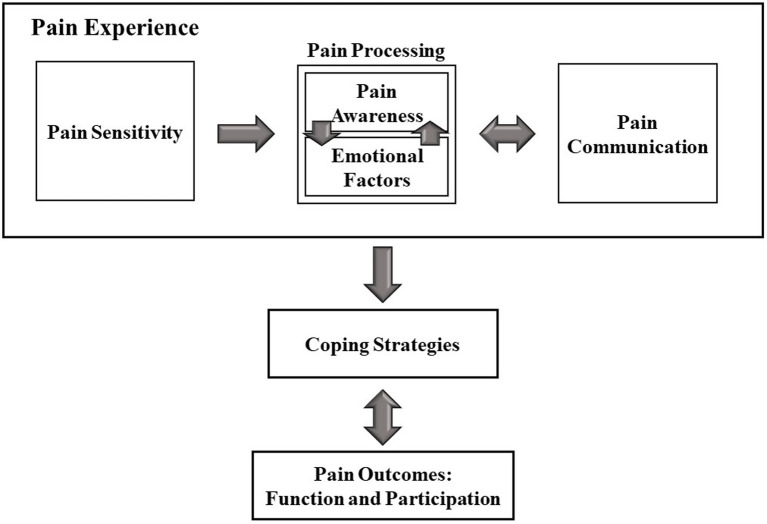
Pain–participation model.

Four primary themes emerged from the data examining the physical pain experience and its implications on daily life in individuals with ASD: (a) the physical pain experience, including the pain sensitivity, pain processing (i.e., pain awareness and emotional aspects of pain), and pain communication; (b) coping strategies, including direct and indirect strategies; (c) function and participation outcomes; and (d) suggestions for Healthcare Providers. The model in Figure 1 demonstrates the concepts that emerged from the analysis and their interrelations, echoing the in-depth interviews with adults with autism. This model strengthens previous literature regarding pain by demonstrating examples from first-hand accounts. Additionally, the model suggests new aspects of pain, which were not mentioned in previous studies, such as pain awareness. The uniqueness and contribution of the model will be further discussed in this paper.

### Pain Experience

All participants (34 citations) defined *pain* as a significant inconvenient, agonizing, and sometimes-traumatic experience. For instance, EK stated, “It is an unpleasant sensation you want to stop; you cannot completely ignore it. When the pain shows up, you immediately notice, it instantly causes discomfort.”

The participants acknowledged the purpose of pain as an indicator that something is wrong, as NC’s words demonstrated as: “Pain, when your body is signaling that something is bothering you, is actually the product of what we feel … when something harms the body, when something is not working as it should.” Some participants emphasized pain’s role as a motivation for action. BG articulated, “Something malfunctions somewhere in one of the systems, so you need to take care of that. Otherwise, [the pain] will get worse.”

Most participants showed a clear understanding of pain’s purpose and function, which helped them accept and cope with it. “Stomachaches, for example, are something I do not understand, but the pain from an injection is much more bearable because I understand its reason” (AH). However, participants also described stress from ambiguous pain, intensified by their need to understand, classify, and identify its causes.

Three main subthemes were recognized in the pain experience context: (1) pain sensitivity, (2) pain processing, consisting of pain awareness and emotional aspects, and (3) pain communication.

#### Pain Sensitivity

The participants described various sensory-sensitivity characteristics: hypersensitivity (six participants), hyposensitivity (seven participants), and neutral (two participants). BF’s and AC’s contradictory reports demonstrated this heterogeneity. BF compared her experience of increased pain sensitivity to others: “Some things that are not painful to others are very painful to me.” On the other hand, AC reported decreased pain sensitivity: “I have pain tolerance. I am less sensitive to pain than most of my friends and family members.”

Some participants who reported having decreased pain sensitivity also addressed their surroundings’ role in shaping their self-perceptions of pain sensitivity. They noted their primary groups saying they were less sensitive to pain. “I know I’m less sensitive to pain because that’s what I’d always been told. My parents, friends, and anyone around me told me I am resistant to pain” (BG). They described this external input from the surroundings as coming throughout their lifespans. TC shared, “When I was a child, I was very sensitive to pain… Today, I am more resistant to pain. I knew that, and my surroundings kept telling me, ever since I was a child.” Like TC’s description, AD reported decreased pain sensitivity: “I think when I was younger, I was more sensitive to pain; today, my resistance to pain is higher.”

Like AD, most participants who reported changed pain sensitivity described a decreased pain sensitivity throughout their lifespan. Only one participant described the opposite (i.e., increased sensitivity along with the lifespan). Relying on the surroundings’ messages as an external input about their own pain experience may result from their difficulties with pain awareness.

#### Pain Awareness

All participants (43 citations) reported uncertainty about the internal pain experience of ambiguous pain. This uncertainty consisted of three layers: the existence, nature, and severity of pain. Only a few participants reported having a fundamental difficulty recognizing the *existence* of pain (i.e., classifying a stimulus as painful). They described being unsure whether the source of the inconvenience they felt was pain or another physical sensation (e.g., hunger, tiredness, or sensory overflow in non-pain sensory modalities). OM shared, “I know that my reading of myself is wrong. Sometimes I feel physical discomfort and do not know if what I feel is from being tired, hungry, or because a strong noise is overflowing my system.”

While only a few participants reported uncertainty regarding the existence of pain, most spoke of challenges defining the second layer, the *nature* of pain. These challenges included identifying the location, intensity, and type (e.g., stabbing, burning, or stinging) of pain: “I do not trust myself when it comes to sensations… If someone asked me where or how much it hurts, I would not know how to answer” (AH). Such experiences demonstrate their challenges during contact with others, especially healthcare providers.

The difficulty in defining pain intensity may be linked to the last layer of uncertainty regarding the pain experience—challenges estimating the pain *severity*. Most participants described these challenges. GM vividly shared her experience: “My leg was caught in the revolving door. I felt a strong pain… I could not understand what my body was signaling to me. Is the pain serious? … I did not know what to do until someone told me I was pale, and the leg did not look as it should. She called an ambulance.”

Given their difficulty characterizing the pain experience, participants often tended not to get treatment or to look first for external information. Many described depending on their surroundings to validate and affirm their pain experiences. AH described: “When I go to the doctor, I ask a lot whether what I am doing is good… I am better off asking than making mistakes. I do not trust myself, my reports, and my sensations and need someone else to tell me I am OK, give affirmation, and describe what I feel.”

The participants reported their reliance on others for pain-related data as simultaneously reassuring and regulating or confusing and frustrating. These positive or negative feelings resulted from the congruence or incongruence with their interpretations of the surroundings and internal experiences. AH gave an example of negative emotions following a doctor’s incongruent interpretation: “I went to doctors on several occasions to understand what I am going through, what is hurting me, and what to do about it, and they did not understand me. It was confusing because the gap between what I felt and what they said I have and the way they treated me was enormous.” On the other hand, NB shared a positive experience following a successful interpretation of her sensations with her friend’s help: “My ear hurt a lot one time. It drove me crazy. I called a good friend, and she helped me understand that the pain is intense and that I should get checked up… I remember the feeling of relief when she articulated my pain.”

These examples reflect the consequences of the participants’ dependency on others to cope with pain awareness difficulties. When others successfully interpret their experience and respond accordingly, this coping strategy may facilitate clarification, resolution, and better self-understanding. However, a mismatch between the internal experience and external response leads to confusion, frustration, and misconception regarding personal pain features.

Another external information source about the participants’ pain was visual input of body-tissue damage (e.g., bleeding, wound, or swelling) and, to a lesser degree, related physical-environment damage (e.g., broken glass, bent car, or torn clothes). NA shared an example: “I was sitting to the side of the bicycle, and my leg got caught in the wheel. I felt something bad had happened, but I did not know what. Then I saw my sandal was completely torn up, and my leg was stuck; only then did I understand I was in pain.”

The participants emphasized the struggle to recognize, interpret, and understand their pain experiences. Interestingly, several attributed pain uncertainties to a general mistrust and misunderstanding of their inner world. NA explained, “I associate the subject of pain perception to social communication. I live my life with the realization and awareness that I misunderstand and misinterpret the world, so I do not trust myself.”

Following the general struggle with understanding and interpreting the outer and inner worlds, participants described the need for external information sources to solve the uncertainty and evoked emotional stress.

#### Emotional Aspects of Pain

Another critical component of all participants’ (38 citations) physical pain experiences was pain’s emotional aspects. When the participants defined *pain*, they addressed two kinds: physical and emotional. “Both include an unpleasant sensation, but each is caused by different triggers, and their manifestation will be different” (AH).

The participants also described that physical and emotional pain often co-occur or ensue sequentially (i.e., emotional aspects serve as triggers and are evoked by physical pain). Many reported that physical pain triggered negative emotions, such as helplessness, distress, and insecurity. AK reflected, “When I feel pain in my body, when something bad hurts me, my anxiety rises. I am in distress.” The participants described that the cognitive, estimated health-and-safety risk level, specifically if life-threatening, also affected their emotional reaction to physical pain. OL explained, “When you realize that the pain will not kill you, it dampens the pain a lot.” Conversely, several participants reported underestimating common and frequent pain (e.g., headaches and superficial injuries) when they understood them to be non-life-threatening: “I know that in most situations when I feel pain, it will not kill me. That is why I do not think much of the stomachaches and headaches I frequently experience” (NA).

The participants described that emotional pain might trigger physical pain. OM echoed many others: “When I feel emotional pain—for example, strong anxiety—I immediately feel it in my body… Emotional pain mostly expresses itself with stomachaches.” Another effect was emotional pain’s amplification of physical pain. “If I am anxious, it does not matter what caused the pain or what kind of physical pain it is, it will be slightly stronger” (NB). As OM’s and NB’s quotations demonstrated, anxiety was a prevalent emotion that might evoke or amplify pain. On the other hand, most participants reported that awareness, knowledge, and familiarity with the painful stimuli promoted better emotional states by providing a sense of control that decreased the physical pain. “In familiar situations, there is more of a sense of emotional calm, and then the physical pain is less strong” (AK).

The participants’ reports indicated a tight bond between the emotional domain and pain experience. This bond was not unidirectional: The emotional aspects of pain and the pain experience concurrently related to each other. Similarly, pain awareness and emotional aspects were reciprocal; both were key factors in pain processing.

#### Pain Communication

All participants (47 citations) reported difficulties expressing pain and attributed them to (1) fundamental communication difficulties, (2) challenges “translating” the internal experience to words, and (3) challenges using prevalent evaluation tools.

Many participants recognized that social interaction deficits deriving from ASD characteristics were a significant factor causing them difficulties expressing their pain and being understood. NB conveyed, “Because of my autism, I usually find it hard to describe what I need or want since it is hard for me to talk to people. When I try to address them, I realize, time after time, that they do not understand me… This manifests when I try to talk to others when I am experiencing pain.”

The participants reported that coping with physical pain, accompanied by emotional reactions, requires effort and tools—leaving little for coping with communication challenges. BG articulated, “I cannot muster the strength and effort needed to express myself [when I am in pain]. It’s always hard for me to communicate, but pain situations make it [more] difficult to apply the strategies and tools I use in my daily communications with those around me.”

Moreover, participants emphasized that physical pain’s abstract nature and internal presence make it harder to define, explain, and share: “I can never explain my feeling of pain. I try … but do not know how to describe it accurately enough: How much it hurts, where, and what kind of pain it is. How can one take a sensation and translate it to words?” (NC).

This challenge of explaining and defining pain with words may link to difficulties using pain-evaluation tools that translate the pain experience into symbols (faces, numbers, colors, etc.). As manifested in AH’s words, this challenge represents the participants’ unique desire and stress to be correct and accurate, which is not simple in the pain phenomenon: “I do not know how to use numbered or colored scales to rate pain; they are too complex. How can I know I’m “translating” my pain correctly to another representation? I struggle with this definition and not being able to confirm that my answer is correct, accurate, and reflects what I feel.”

### Coping Strategies

This study revealed two coping-strategy types: (a) direct strategies to solve the pain situation and (b) indirect coping strategies to ease the pain experience. The latter type was reported as more commonly used.

#### Direct Coping Strategies

##### Preference to Cope With Pain by Oneself

Given their significant challenges in communicating pain, it is understandable that all participants (24 citations) reported preferring to cope with pain by themselves, as AH’s description demonstrated: “I have difficulties reporting pain… When I tried to reach out for help, … I could not get the response I needed. I realized that I had to get by on my own. Bit by bit, … I simply rule out going to others in the context of pain.”

Many participants described depending on others’ help when in pain as children. Two parallel processes throughout the years contributed to their preference for coping without external assistance. The first process was maturation, gradually self-learning their pain characteristics and mechanisms and transitioning to more independent coping. For example, NB stated, “As a child, the sole responsibility for solving painful situations was my parents.’ Today, I better understand where the pain is coming from, its side effects, and how to treat it.”

The second process was a form of mistrust of others’ assistance during pain situations. Repeated experiences of being misunderstood and trying and failing to get help created a chasm between the participants and their surroundings: “Because our experiences with trying to get help are painful, we do not want to ask for help from those around us when in pain… The outside is not a place that lends help… Only I understand my pain. Only I can help myself” (BF).

The participants reported asking for help only when they evaluated the pain as severe—intense, long-lasting, or accompanied by external signs of a serious injury. OM described such an experience: “I did not tell anyone … that I fell or was in pain. After several days, my leg swelled a lot, so I said to myself, ‘OK, maybe I should tell my parents that I’m feeling pain.’” As reflected in OM’s report, participants agreed that seeking others’ help would be the last resort.

##### Asking Others for Help

When participants did ask for help, most (27 citations/12 participants) reported asking family members (mainly parents) or doctors. Several also asked friends, partners, or housing facility assisting staff. Most participants identified their parents as significant figures in pain situations, helping them define the pain situation, look for solutions, and bridge the gap with medical professionals:

If I need help when I’m in pain and not feeling well, I go to my mother. When I’m not making sense to others or even myself, my parents understand me. They know how to help me understand what’s happening to me and solve the problem. They also help me communicate my needs to staff members who will take care of me. (NA)

Although comprehensive agreement on the positive value of approaching parents for help was apparent, asking medical professionals for help was controversial among the participants. Some shared negative interactions with medical staff, which included inadequate assessment and treatment of physical pain, resulting in medical complications. OM’s experience is one example:

I told the doctors in the emergency room that I was in great pain. They did their tests and finally said, “You can go home,” like everything was fine. I kept telling them I was in pain, but they discharged me anyway. My mother [took] me back to the hospital several hours later… I had to be hospitalized immediately because of a severe leg infection that already started spreading.

The participants’ shared experiences suggested three distinct triggers for inadequate treatment of physical pain. The first they described as *disrespect*, feeling the medical staff did not take their complaints seriously. “When I seek help and say to the doctors, ‘I am in pain,’ I expect them to pay attention… But what happens is that when I go to the doctors, they do not take me seriously. I had the feeling of being really disrespected” (GM).

The second trigger is the medical staff’s *misunderstandings* due to communication difficulties: “They do not get me, do not understand what is happening to me, why I came, and what I need. I go seeking help, but during the appointment, I realize time after time that it is not going to work, and I give up. I know their treatment of me will not be adequate because they do not understand me” (NB).

The last trigger mentioned was *conflicting expectations* regarding the role of each side in the interaction. Whereas the medical staff expects the person with ASD to articulate their complaints and requests, the person with ASD copes with an initiation challenge and expects the medical staff to identify the proper treatment:

After I broke my leg, I was in a lot of pain but wasn’t offered painkillers. I thought it was the doctor’s or the nurse’s role to offer me medication, but they did not do that. I kept suffering excruciating pain. Today, I realize they expected me to tell them … to ask them. But it’s hard to address someone and suggest my own ideas. (AK)

BF described a similar incident. “On the third day of the hospitalization (post-hysterectomy), the nurse suddenly came over and asked, ‘What are you getting for the pain?’ I told her I was not receiving anything. She asked why I had not asked for anything.”

The participants’ experiences emphasized the gap between their needs and expectations and the medical staff’s prevalent attitude. AK’s and BF’s words indicating this gap also demonstrated an aspect of the complex relationship of people with ASD to pain medication. They shared that their difficulty initiating communication aimed at receiving care resulted in less or no pain-medication treatment.

##### Using Pain Relievers (Analgesics)

Participants described asking for analgesics as a challenge stemming from their difficulties initiating communication. Analgesic use appeared to be a broader subtheme (33 citations/13 participants). Participants described preferring to avoid analgesics due to concerns over (1) using chemical substances, (2) masking internal information, and (3) inability to report pain accurately to the medical staff due to the analgesics’ influence. Many participants described hesitating to use analgesics because they worried about the chemicals’ effects on their bodies. TC shared, “I do not like to put chemical substances into my body. My body chemistry is challenged as it is. I fear the effects that painkillers have on my body.”

Another common concern was masking internal sensations (i.e., losing pain input, such as severity, intensity, and location), which might decrease pain awareness. RS said, “The pain from the hernia was getting much worse, so I went to the hospital. I was in a lot of pain but afraid to ask for painkillers. I feared I would lose my awareness of the pain and then lose control over my body.”

Participants also mentioned losing internal information after using analgesics in conjunction with their third concern—the inability to communicate with and get help from medical staff. They described fearing situations in which the medical staff would ask about the nature of their pain, and they could not answer because of the analgesic effect—and fearing misdiagnosis as a result. GM shared her emergency room experience after she broke her leg: “I did not want to ask for pain pills. I was afraid that if I took them, then when the doctor came, I would not be able to tell him what I was going through exactly. I have to rely on information from my leg for that. So, I thought to myself, I will not take painkillers … so no misdiagnosis would occur.”

Despite their reluctance, a few participants reported using analgesics for intense or unpredictable pain. EK shared her menstruation experience: “For example, when I’m on my period, I cannot control it… I mean, it’s strong waves of pain you do not know how or when they’ll appear. When I … recognize that unpleasant sensation is beginning, I immediately take pills. I do not wait for it to start hurting.”

#### Indirect Coping Strategies

The participants’ reports indicated less prevalent use of direct pain-related coping strategies, including asking for help and using analgesics, than indirect pain-related coping strategies. These included ignoring and resting, engaging in alternative activities, covering/holding the sore area, and self-injurious behavior (SIB).

##### Ignoring and Resting

Many participants (19 citations/11 participants) mentioned ignoring the pain as a helpful strategy in mild pain situations. They reported continuing their daily routines, hoping their pain would dissipate. AC described, “I try to pass the time and let the pain fade.” Many participants also reported resting as an opposite strategy. They described a definite stop in functioning that enabled them to relax their bodies by sitting or lying in bed. NC elaborated, “When I’m in a lot of pain or hurt in a lot of places, … I just lie in bed. I try to relax my body, rest. When my body is relaxed, the pain decreases.” Some participants described combining other relaxation techniques with resting, such as meditation, deep or yogic breathing, listening to constructed meditations, and repeating health-focused mantras (e.g., “I feel good,” “My body is strong”).

##### Engaging in Alternative Activities

Along with relaxation techniques, participants (26 citations/14 participants) described various alternative activities that served as coping strategies. The common denominator was that these were favorite, preferred distracting activities. The activity reported most often was using a cellphone for gaming, watching videos, and browsing social media. OL shared, “When I am in pain, … the best place to escape to is my phone. There are many activities I can distract myself with—movies, games, and more. It’s as if it passes the pain, and you do not think about it.” Other activities participants mentioned were talking with someone, reading a book, watching movies, taking a shower, thinking, and daydreaming.

Whereas participants repeatedly described preferring to engage in purposeful activity, a few expressed engaging in emotional activities (e.g., crying or complaining) to cope with pain. Although possible and familiar, such a coping strategy was not prevalent or preferred, as OM’s words demonstrated: “I rarely cry because of pain. I tend to complain when I need to unload. When I was sick or went to the ER, I saw many people crying. I guess it’s good to cry, but I do not tend to that. It’s uncommon for me, but I can remember several times when it happened.”

##### Covering/Holding the Sore Area

Several participants (16 citations/7 participants) reported a specific alternative activity subtype that included covering, holding, or massaging the painful area. These behaviors reflect the spectrum of strategies from ignoring to actively coping with pain. On one side, several participants reported covering the painful area to avoid visual signs that may remind them of their pain. AC gave an example: “A large rock fell on my leg and wounded me. The wound bled. I preferred not to pay attention to the wound; ignore it and keep working. I pulled my pants down to cover the wound so I did not see and think about my pain.”

Whereas covering the painful area aimed to ignore the pain, participants described holding the area as deriving from acknowledging the pain’s existence. They explained that this behavior aimed to protect the painful area and dampen the pain. AK described, “At the end of the climb on the mountain, my hands were full of scratches. They burned and hurt. I remember holding them close to my body. I wanted them to be protected and no one could touch them. The pressure on them, the contact, lessened the pain.” Like AK, several participants referred to touch, specifically deep pressure massage, as a pain-coping strategy. Most reported massaging the painful area to dampen the pain; one described massaging another area simultaneously as a distractive stimulus.

##### Self-Injurious Behaviors

Several participants (14 citations/five participants) reported another strategy to reduce pain was self-injurious behaviors (SIB), harming oneself while in pain. The SIB severity varied between mildly (e.g., pinching, hair-pulling, and scab-picking) and severely injurious (e.g., head-banging, starting a physical confrontation, and suicide attempts) behaviors. AK’s comment manifested mildly SIB at one extreme: “Sometimes, when I am in a lot of pain, I pinch myself somewhere else. Then the other pain distracts me from the sharp pain.” At the other extreme, BG described severe SIB: “During high school, when I was in pain, I would bang my head on the wall… One time I was waiting for a doctor’s appointment, and my head really hurt, a strong migraine. I came close to my parents and banged my head on the wall next to them. It calmed me.” The participants’ reports demonstrate varying SIB severity and function. In AK’s case, the SIB created a distraction to reduce pain or perhaps served as an unconscious way to induce the “pain inhibits pain” mechanism. In BG’s case, the SIB was carried out for emotional relief.

### Implications of Pain on Function and Participation

All participants (34 citations) described pain’s implications on function, ranging from a complete stop in daily function to full and uninterrupted function. Some participants firmly stated they could not function when in pain because of its intense influence on them. Others reported continuing to function as usual without changing their daily routine. Most participants described more moderate implications of pain on their function. They reported needing to reduce the amount and rate of their daily functions when in pain. “I have to decide what to spend my energy on. I cannot perform all my functions as usual; I must decide which are especially important, and these I do” (NC). Many participants reported skipping or reducing their engagement in daily functions, including work [“Long-lasting pain interrupts my concentration, it will make me miss work” (AK)], education [“On days when I am on my period, I’m in so much pain, I cannot participate in classes” (GM)], and leisure [“I cannot do even things that I really enjoy, like playing music” (AD)].

Another pain-related implication participants described was involvement in bondage and discipline, dominance and submission, sadism, and masochism (BDSM). Being a part of a BDSM community expressed their sexual functioning and social participation. Of the three participants who mentioned engaging in BDSM, two were a couple, and the third was not in a stable relationship. Although these participants differed in their reported pain thresholds (hyper-/hyposensitivity), they all related to the sense of control over pain as a source of enjoyment and pleasure during BDSM interactions.

BG described her feelings from the “dominant” role viewpoint: “I’m on the side that likes to cause others pain… I like that I can hurt others without experiencing pain myself, be in control. It also allows me a chance to see how pain transforms from a bad thing to something good. That pleases me.” Her partner, RS, shared his feelings from the “submissive” viewpoint: “I personally enjoy being on the controlled side. The pain I feel … is pleasant… When I choose to engage in a BDSM session, I know I’m about to feel pain… Readying myself for pain and being able to control the pain I am about to feel has some effect.” GM also reported satisfaction from the sense of control over the pain she acquires in the submissive role:

I do not necessarily love pain, but I know I can stop it when it is too much. It’s controllable, and no one can hurt me if I do not want it. Suddenly, the pain becomes pleasant. It’s mostly giving the other side what it needs… Most times, I find it pleasant for me, too. It provides me a safe place where I can experience pain in a pleasant and controlled way, and I get what I want at the same time—it can be sex, and it can be a hug.

### Suggestions for Healthcare Providers

The participants suggested ideas to promote a better-suited healthcare experience in pain situations. The participants’ suggestions addressed various needs and challenges they cope with when seeking health care. These suggestions included theoretical issues such as healthcare providers’ attitudes and practical ideas, such as visual aids or environmental accommodations. Their ideas are presented in Table 2.

**Table 2:** Suggestions for healthcare providers.

Need	Participant suggestion
Communication Mitigation	“I need the option to communicate differently, not just by speaking. If the doctor had shown me a picture of the human body and told me, ‘Show me where it hurts,’ it would have been a lot easier to answer compared to just asking, ‘Where does it hurt?’” (OK).
Initiation Responsibility	“I do not usually ask for help; I need the doctor to be the one to tell me, ‘We have something to give you for the pain’ and suggest the option more than once. I am not used to asking for help; it is hard for me to initiate asking for help” (RS).
Emotional Support	“Emotional support, attitude and personal connection are critical to me. I need to feel safe. The doctor wants what is best for me and is attentive to me” (NC).
Sensory Modifications	“The ER is loud; there are screams and bright lights. I cannot speak about my pain or answer questions in such an environment. It would help to have a private room without much light and noise. Then, I would be able to self-reflect, think, and communicate” (BF).
Information Processing	“It would help me if there were booklet with structured sentences and demonstrative pictures so I could understand what I suffer from, what am I going through, what I am experiencing and followed by a description of my situation” (NB).

## Discussion

This study examined the physical pain experiences that adults with ASD shared. Its phenomenological approach captured first-hand accounts of the pain experience through their lens. It spotlights the crucial role of pain awareness and communication in their daily lives beyond the previous literature, which focused on aspects of pain such as neural response (e.g., Failla et al., 2018), sensitivity (e.g., Failla et al., 2020), and behavioral expression (e.g., Nader et al., 2004). Thus, little was known about the pain experience of people with ASD in their daily life contexts.

This study’s results suggest a novel theoretical model (Figure 1) reflecting the participants’ insights into the sequence and implications of the physical pain experience. The sequence begins with physical pain, consisting of pain sensitivity, awareness, emotional aspects, and communication. As a result of this experience, the person with ASD chooses and applies coping strategies. The outcomes of this process manifest in function and participation levels. The model contributes a constructed conceptualization of the pain experience in people with ASD and its consequences.

### Pain Experience

#### Pain Sensitivity

The unique sensory characteristics of people with ASD have many layers, including pain sensitivity. Three aspects of pain sensitivity emerged from the data: (1) its characteristics, (2) the surroundings’ input regarding it, and (3) changes in perceived sensitivity across the lifespan. This study revealed variable pain-sensitivity characteristics among participants. Several participants reported a gradual change in pain sensitivity across the lifespan; most described an *increased* pain threshold. Many of those who defined themselves as less sensitive to pain shared that their primary group (mainly parents) told them over the years that they had higher pain thresholds than others.

Sensory sensitivity has long been researched in this population using various methodologies, mostly parental reports (Moore, 2015). Previous research demonstrated inconsistent findings regarding pain sensitivity in people with ASD, often depending on the chosen methodology. Generally, studies based on physiological measurements indicated no pain-threshold difference between people with and without ASD (Allely, 2013). Psychophysics-based studies have indicated hypersensitivity to supra-threshold pain stimuli (Failla et al., 2020). In contrast, studies based on case studies and self- or parental reports pointed to pain hyposensitivity (Allely, 2013). Neuroimaging studies aiming to characterize pain sensitivity in people with ASD also were inconsistent. For example, whereas Gu et al. (2018) reported pain hypersensitivity, Vaughan et al. (2020) reported pain hyposensitivity. The first-hand accounts in this study align with the inconsistent literature, representing diverse pain-sensitivity levels: pain hyposensitivity, pain hypersensitivity, or the same sensitivity as others (neutral).

The literature debated whether pain sensitivity changes across the lifespan. Lu et al. (2007) found that greater age predicted higher pain tolerance, lower pain intensity, and pain unpleasantness in neurotypical children and adolescents. These results align with this study’s participant reports about perceived pain sensitivity changing from childhood to adulthood. On the other hand, Riley et al. (2000) found no changes in pain intensity or pain unpleasantness but age-related changes in emotional pain-related distress and pain behaviors in chronic pain adult patients. They attributed the changes to attitudes, beliefs, and life circumstances. These findings may link to this study’s third pain sensitivity subtheme—the influence of others’ input on the perceived sensory sensitivity. Riley et al.’s study strengthened our hypothesis that external input from the surroundings affects individuals’ perceptions of their pain sensitivity by shaping attitudes and beliefs.

Despite the inconsistent literature on pain sensitivity, there is a prevailing belief, frequently based on anecdotal observation or clinical impressions, that pain insensitivity is common among people with ASD (Messmer et al., 2008). Because of this prevalent belief, people with ASD may be subject to mistaken input by their surroundings regarding their pain sensitivity. The study of Nader et al. (2004) provided evidence of misinterpreting the pain experience of people with ASD. They found incongruencies between parents’ reports of their children’s pain and observed pain responses as interpreted by professionals during an invasive procedure. One cause of the prevailing misconception regarding pain sensitivity in people with ASD is their core characteristic of communication difficulties, manifested as different social-communicative behaviors during pain episodes (Summers et al., 2017). People with ASD also may exhibit confusing behaviors such as SIB, which might be interpreted as pain hyposensitivity (Symons, 2011).

Although pain is subjective by nature (Love-Jones, 2019), participants elaborated on their frequent need to rely on others, especially their parents, to learn and determine their various pain characteristics and did not mention specifically the level of their pain sensitivity.

#### Pain Awareness

All participants reported challenges in pain awareness as a form of uncertainty regarding the pain’s existence and nature (i.e., location, intensity, and type). Their difficulties recognizing, interpreting, and characterizing pain may result from interoception challenges—the afferent signaling, central processing, and neural and mental representation of internal bodily signals (DuBois et al., 2016; Critchley and Garfinkel, 2017). Some models expand the interoception definition to encompass motivationally important physiological signals, including pain (Craig, 2002). Abnormal interoception has been found among people with ASD, with a slight tendency for hyporeactivity in interoceptive awareness (DuBois et al., 2016). Contemporary models differentiate three interoception dimensions: (a) accuracy, the precision of detecting internal body sensation, (b) sensibility, the self-perceived tendency to be internally self-focused and interoceptively aware, and (c) awareness, the metacognitive awareness of interoceptive accuracy (Garfinkel et al., 2015). In this study, participants reported challenges in recognizing, interpreting, and characterizing pain sensations, reflecting difficulties with the first interoception dimension (accuracy). In contrast, their ability to acknowledge those difficulties reflects preserved interoceptive awareness. These findings are congruent with the study of Garfinkel et al. (2016), which indicated impaired interoceptive accuracy alongside intact interoceptive awareness in people with ASD.

The participants’ reports may also point to another aspect of interoception, *interoceptive impact*, which was suggested by Dunn et al. (2022). The interoceptive impact is the influence of interoception on everyday life (Dunn et al., 2022). The participants described challenges in pain awareness and accompanied those descriptions with daily function manifestations. For example, some participants described challenges in recognizing pain, which affected their ability to identify the source of their distress and, as a result, to adjust the level of daily functioning (e.g., using a broken limb). The current study strengthens the concept of interoceptive impact and recognizes it as a 4^th^ dimension of interoception.

The participants’ reports in this study echoed a significant challenge with pain awareness, ascribing it as the primary source of their difficulty regarding the pain experience. They described the lack of internal information as disturbing, triggering a sense of uncertainty, and depending on external information from people around them or visual input to resolve this internal inconvenience. When external information is congruent with their inner pain experience, it results in reassurance and regulation; incongruences result in confusion and distress.

#### Emotional Aspects of Pain

The participants addressed two pain categories, physical and emotional, which appear simultaneously or sequentially. The participants elaborated on the significant effect of their emotional state on their pain experience. The International Association for the Study of Pain defined *pain* as involving actual or potential tissue damage or as the individual describes such damage (Raja et al., 2020). A growing body of literature suggests emotional stimuli may provoke pain like that associated with acute physical pain (Frumkin et al., 2020). The emotional responses to pain, particularly fear, might be more critical than physical pain in determining the suffering the pain causes and affect disability and performance levels (Crombez et al., 1999). The findings of Crombez et al. (1999) support this study’s results, emphasizing the significant role of emotional aspects in the pain experience. Participants addressed two main factors that affect their fear of pain: (1) the sense of control over the physical pain and (2) the extent of life risk associated with the pain. These factors are cognitive-driven and based on knowledge, familiarity, and awareness.

Interoception enables top-down, predictive, multisensory integration and body ownership (DuBois et al., 2016). Seth (2013) suggested that the gap between incoming interoceptive signals and predictive efferent signals is a base for anxiety. Due to interoception deficits, people with ASD may encounter uncertainty in physical pain experiences and often cope with intolerance to uncertainty. This intolerance motivates them to desire predictability and actively seek certainty. When the uncertainty remains unsolved, it may evoke *uncertainty paralysis* (i.e., a feeling of being stuck; Stark et al., 2021). This paralysis may explain the tendency to stop functioning in pain that participants described in several contexts.

In this study, many participants reported or manifested rational and binary thinking to cope with uncertainty in the pain experience. The participants’ evaluation of the extent of life risk associated with the pain prominently demonstrated such dichotomous thinking. Dichotomous thinking expresses cognitive rigidity, an ASD core characteristic (American Psychiatric Association, 2013) associated with anxiety in children with ASD and directly mediated by uncertainty intolerance (Ozsivadjian et al., 2021).

### Pain Communication

The participants provided an extensive description of difficulties communicating pain. They attributed these difficulties to (a) core communication deficits, (b) challenges conceptualizing pain, and (c) challenges using prevalent pain-evaluation tools. It has long been recognized that pain is a physical sensation and a complex social experience, and social communication plays a crucial role in its expression (Craig, 2009). People with ASD are likely to encounter challenges reporting pain due to their core difficulties in social communication, including failure to initiate or respond to social interactions and deficits in nonverbal communication (American Psychiatric Association, 2013). These difficulties hinder their ability to accurately report the degree and nature of their pain experience (Failla et al., 2021).

Another potential source of the difficulty participants mentioned is their challenges conceptualizing the abstract pain experience into words or symbols. Prior studies found that people with ASD had weaker conceptual reasoning ability than neurotypicals of similar ages and cognitive abilities (Williams et al., 2014). Moreover, pain conceptualization requires observing oneself and others (Loeser, 1996), which often challenges people with ASD. Two deficits affect their ability to consolidate the pain concept: (1) deficits in empathy and theory of mind, affecting their understanding of another’s mental state (Baron-Cohen, 2000) and (2) difficulty in pain interoception, affecting their ability to notice and characterize their pain experience.

The last source of pain communication difficulty specifically affects their ability to express their pain to health care providers—challenges in using pain-evaluation tools. People with ASD may encounter barriers to reliable reporting due to decreased communication or cognitive abilities. Pain typically is measured by verbal self-reports, considered the “gold-standard” pain assessment (Failla et al., 2021). This study’s participants specified difficulties related to the facial, numerical, or color pain representations often used to assess pain medically. Pain also is measured by observation-based assessments (Moore, 2015; Failla et al., 2018) that code nonverbal responses (e.g., facial expressions) to stimuli. However, these responses may be atypical in people with ASD (Failla et al., 2018). Previous studies demonstrated mixed findings on facial reactivity in children with ASD undergoing painful procedures compared to non-ASD populations (Nader et al., 2004; Rattaz et al., 2013).

The challenges in pain awareness and core communication symptoms in people with ASD result in major difficulties expressing their pain experience to others, particularly health care providers. Communication difficulties may result in providers misunderstanding the pain characteristics, such as location and intensity, and affect their ability to diagnose the pain source adequately. In this study, several participants described pain mis/underdiagnoses that resulted in severe medical complications.

### Coping Strategies

Pain researchers have studied coping with pain thoroughly (van Damme et al., 2008). However, studies of the pain experience in people with ASD focused on understanding pain as a phenomenon in this population and defining its features (e.g., Allely, 2013; Moore, 2015). Little is known about pain-coping strategies among people with ASD.

#### Direct Strategies

Direct coping strategies reflect a preference to cope with pain by oneself. Participants who preferred to cope with pain without depending on others, as they had in childhood, attributed this change to two main reasons: maturation and independence, including enhanced skills and coping strategies, and mistrust of others. They reported enhanced skills improving their ability to cope with pain. These reports agreed with the longitudinal research of Shattuck et al. (2007) on adolescents and adults with ASD, indicating that most of their sample experienced declining ASD symptoms and maladaptive behaviors. The adolescents exhibited more significantly improved reciprocal social interactions; the adults improved most on restrictive, repetitive behaviors. Such modifications across the lifespan can explain the higher capacity for more independent coping with pain.

Another reason our participants preferred coping with the pain alone was their mistrust of others. Previous studies indicated higher suspicion or mistrust levels in people with ASD (Blackshaw et al., 2001). Repeated adverse social interactions may exacerbate social anxiety (Kuusikko et al., 2008), leading to social withdrawal (Ding et al., 2019). This progression of repeated adverse incidences of disappointment may explain their preference not to get assistance from others.

Concurrent with the notion that people with ASD experience mistrust others, participants reported asking for help as a last resort. They described reaching out, mainly to their parents, for help in evaluating the pain situation, seeking a solution, and mitigating miscommunication with health professionals. Asking health professionals for help was inconsistent, often accompanied by inadequate evaluation and treatment. Similarly, Ely et al. (2016) conducted a qualitative study of pain communication in children with ASD and found the primary support source was the children’s parents.

Despite the significance of external support in pain situations among people with ASD, many participants in this study reported negative interactions with health professionals. Negative pain-related interactions constitute adverse social interactions that can detrimentally influence a person’s sense of well-being, life stress, supportive networks, and psychological distress in people with chronic pain (Fernández-Peña et al., 2020). Studies have associated satisfaction with support as leading to both adaptive and maladaptive coping, but disappointment to only maladaptive coping (Holtzman et al., 2004).

Previous research reflected the communication gap between people with ASD and health care professionals, resulting in overlooking pain and inadequate treatment for this population (Moore, 2015). Health care professionals reported lacking knowledge and training regarding people with ASD and thus low self-efficacy in managing their medical care (Walsh et al., 2020). Women with ASD reported more significant health care challenges, including anxiety, emotional distress relating to communication, and anxiety about waiting rooms. They also noted self-disclosure of diagnostic status and lack of ASD awareness by health care professionals (Lum et al., 2014).

Using analgesics is a prevalent coping strategy (e.g., Barry et al., 2004). However, the literature has associated it with long-term increased pain, disability, and poorer psychological adjustment to pain (Jensen et al., 1991; Snow-Turek et al., 1996). This study’s participants widely shared their challenge in using analgesics, their concerns about consuming chemicals and their fear of masking internal information that may influence their ability to report pain to the medical staff. In the study of Rattaz et al. (2013), neurotypical children received local anesthetic or sedation almost systematically before a medical procedure, but less than half the children with ASD received the same treatment. Rattaz et al. suggested that health professionals might be less attuned to pain alleviation in children with ASD.

These findings indicate that people with ASD have more responsibility for initiating and requesting analgesics than their neurotypical peers. On the other hand, this responsibility clashes with their concerns about using analgesics and core deficits in initiating communication.

#### Indirect Strategies

Many participants in this study mentioned ignoring the pain—behaving as if there is no pain—as an effective coping strategy (Peres and Lucchetti, 2010). Children with ASD have reported using this coping strategy, avoiding talking about the pain and redirecting the conversation to other interests (Ely et al., 2016). Previous studies with chronic pain patients found inconsistent results regarding whether ignoring or avoiding pain is an adaptive coping strategy. Some studies indicated that ignoring pain sensations predicted lower perceptions of control over pain (Haythornthwaite et al., 1998) and higher depression levels (Haythornthwaite et al., 2003). Others associated it with better pain outcomes, such as more acceptance and lower pain-related anxiety (McCracken and Eccleston, 2003).

Whereas ignoring pain is a form of active engagement and effort, resting represents coping by a complete stop from daily functioning. Resting has been associated with increased pain and disability in people with chronic pain (Jensen et al., 1991; Tan et al., 2001; Samwel et al., 2006). Both ignoring and resting were found to have negative implications. These results suggest that an extreme coping strategy, whether an intense activity or a complete stop, is ineffective and has adverse effects.

Some participants described combining resting with relaxation techniques such as mantras and breathing. Previous research regarding relaxation techniques for pain was insufficient due to methodological inadequacies (Smith et al., 2018). Despite the lack of rigorous evidence regarding the use of relaxation techniques in general, positive pain-related mantras were well established as a subset of the self-statements coping strategy. *Self-statements* are a set of phrases individuals internally rehearse to cope with pain experiences (Fernandez, 1986). Self-statements predict greater perceptions of control over pain and self-efficacy in people with chronic pain (Haythornthwaite et al., 1998). It has been suggested that using self-statements recruits the ASD characteristic of repetitive behavior (American Psychiatric Association, 2013), redirecting it into an effective pain-coping strategy.

This study’s participants also commonly reported engaging in alternative distracting activities and thinking to redirect their attention from the pain (Peres and Lucchetti, 2010). Advantages and disadvantages of distracting oneself from pain have been found in people with chronic pain. On the one hand, being involved in an alternative activity is related to lower pain sensations, a more positive mood, and decreased pain catastrophizing. On the other hand, it was associated with more pain-related anxiety and less healthy function (Haythornthwaite et al., 2003; McCracken and Eccleston, 2003; Peres and Lucchetti, 2010).

Involvement in alternative activity as a distractor from pain ranged from active (e.g., taking a bath) to passive (e.g., listening to music) distraction. The study of Dahlquist et al. (2007) showed that both active and passive distractions increased pain tolerance and thresholds relative to the baseline in neurotypical children, but active distraction was significantly more effective.

The participants in this study mentioned several unique behaviors linked to the painful area: holding, massaging, and covering. These behaviors may serve as pain-coping strategies to protect the affected body area or minimize pain through mechanisms associated with tactile stimulation or increased circulation (Sullivan et al., 2004). Covering a sore area has decreased pain ratings (Vijayan et al., 2015). These results demonstrate that pain perception depends on multisensory body representations. Hence, a change in pain-related sensory modality (e.g., vision) might reduce pain and be utilized as a pain coping strategy.

Similarly to reporting behaviors related to the painful area, several participants reported using SIB to reduce pain. Previous studies regarding SIB indicated that SIB might relate to untreated pain caused by a medical condition, such as painful digestive or skin problems (Richards et al., 2016). In addition, researchers and theoreticians suggested that SIB may serve as a pain-coping strategy, proposing that individuals engage in SIB to release endogenous opiates, which results in feeling pleasure (Sandman, 2009) and pain relief (Holden et al., 2005).

### Function and Participation

The participants reported a wide span of pain implications on function. The effect can be arranged on an extreme spectrum from “freeze” to “function.” Some participants described a complete break from their daily routine, whereas others reported full function alongside the pain. The most affected functions were work, education, and leisure.

Previous research in children with ASD revealed that increased pain sensitivity affects other physiological functions (e.g., sleep and gastrointestinal function) and decreases participation in daily activities (Riquelme et al., 2018). Research among people with chronic pain demonstrated vast implications for their social participation and daily function, including family life, leisure, and work (Dueñas et al., 2016).

Participants described engaging in BDSM activity and being a part of the associated community as another implication of the pain experience for participation. They related to the feeling of controlling pain as a source of pleasure. These reports align with the qualitative study of Dewinter et al. (2017) of BDSM in ASD, describing a sense of agency relating to sexual interest and arousal.

### Suggestions for Healthcare Providers

During the interviews, the participants themselves suggested ideas may promote a better-suited healthcare experience in pain. These suggestions were various, practical, and theoretical. The first-hand accounts and the suggestions deriving from them may help build a bridge for healthcare providers toward a more adequate, efficient, and accurate assessment and intervention in pain situations of people with ASD.

## Data Availability Statement

The original contributions presented in the study are included in the article/supplementary material; further inquiries can be directed to the corresponding author.

## Ethics Statement

This study involved human participants. It was reviewed and approved by the University of Haifa Ethics Committee of the Faculty of Social Welfare and Health Sciences. The participants in this study provided their written informed consent to participate in this study.

## Author Contributions

MK-L contributed to conceptualizing, collecting data, analyzing, interpreting, and writing the original draft. NS contributed to conceptualization, consulted regarding the qualitative methods, reviewed, edited, and assisted in interpreting the results. YG, TB-S and IW-F administrated the project, reviewed and edited. TH assisted in sample recruitment. EG supervised, contributed to conceptualization, reviewed, edited, and assisted in interpreting the results. All authors contributed to the article and approved the submitted version.

## Conflict of Interest

The authors declare that the research was conducted in the absence of any commercial or financial relationships that could be construed as a potential conflict of interest.

## Publisher’s Note

All claims expressed in this article are solely those of the authors and do not necessarily represent those of their affiliated organizations, or those of the publisher, the editors and the reviewers. Any product that may be evaluated in this article, or claim that may be made by its manufacturer, is not guaranteed or endorsed by the publisher.
